# Integrated Reanalysis of Global Riverine Fish eDNA Datasets Shows Robustness and Congruence of Biodiversity Conclusions

**DOI:** 10.1111/mec.70340

**Published:** 2026-04-17

**Authors:** Yan Zhang, Heng Zhang, Hiroshi Akashi, Camille P. Albouy, Kara J. Andres, José Barquín, Jeanine Brantschen, Richard E. Connon, Joseph M. Craine, Deirdre Gleeson, Alejandra Goldenberg‐Vilar, Alexia M. González‐Ferreras, Chelsea Hatzenbuhler, Kamil Hupało, Josephine Hyde, Wataru Iwasaki, Mark D. Johnson, Aron D. Katz, Vyacheslav V. Kuzovlev, Courtney E. Larson, Laurène A. Lecaudey, Florian Leese, Matthieu Leray, Feilong Li, Till‐Hendrik Macher, Quentin Mauvisseau, María Morán‐Luis, Georgia Nester, Helio Quintero, Tsilavina Ravelomanana, Merin Reji Chacko, Mattia Saccò, Naiara Sales, Tamara Schenekar, Martin Schletterer, Saskia Schmidt, Nicholas O. Schulte, Robin Schütz, Jinelle H. Sperry, Emma R. Stevens, Sarah A. Stinson, Steven Weiss, Fei Xia, Hui Zhang, Song Zhang, Wenjun Zhong, Shuo Zong, Loïc Pellissier, Xiaowei Zhang, Florian Altermatt

**Affiliations:** ^1^ State Key Laboratory of Water Pollution Control and Green Resource Recycling, School of the Environment Nanjing University Nanjing China; ^2^ Department of Aquatic Ecology, Eawag Swiss Federal Institute of Aquatic Science and Technology Dübendorf Switzerland; ^3^ Department of Evolutionary Biology and Environmental Studies Universität Zürich Zürich Switzerland; ^4^ Department of Natural Sciences, Faculty of Arts and Sciences Komazawa University Setagaya Japan; ^5^ Ecosystems and Landscape Evolution, Institute of Terrestrial Ecosystems, Department of Environmental Systems Science ETH Zürich Zürich Switzerland; ^6^ Unit of Land Change Science, Swiss Federal Research Institute for Forest, Snow and Landscape Research (WSL) Birmensdorf Switzerland; ^7^ Department of Ecology and Evolutionary Biology Cornell University Ithaca New York USA; ^8^ School of Biological Sciences Illinois State University Normal Illinois USA; ^9^ IHCantabria—Instituto de Hidráulica Ambiental de la Universidad de Cantabria Santander Spain; ^10^ Center for Ocean Solution, Hopkins Marine Station Monterey California USA; ^11^ School of Veterinary Medicine University of California, Davis Davis California USA; ^12^ Jonah Ventures, 5485 Conestoga Ct STE 210 Boulder Colorado USA; ^13^ UWA School of Agriculture and Environment The University of Western Australia Perth Australia; ^14^ SpecPro Sustainment and Environment, c/o Office of Research and Development, United States Environmental Protection Agency Duluth Minnesota USA; ^15^ Aquatic Ecosystem Research, Faculty of Biology University of Duisburg‐Essen Essen Germany; ^16^ Aquatic Ecology, Faculty of Biology University of Duisburg‐Essen Essen Germany; ^17^ Biodiversity and Conservation Science Department of Biodiversity, Conservation and Attractions Kensington Australia; ^18^ Biologic Environmental Survey East Perth Australia; ^19^ Department of Integrated Biosciences, Graduate School of Frontier Sciences The University of Tokyo Chiba Japan; ^20^ Engineering Research and Development Center Champaign Illinois USA; ^21^ Department of Entomology University of Illinois at Urbana‐Champaign Urbana Illinois USA; ^22^ Faculty of Environmental Management and Ecology Tver State Technical University Tver Russia; ^23^ Tver Center for Hydrometeorology and Environmental Monitoring Russian Federal Service for Hydrometeorology and Environmental Monitoring Tver Russia; ^24^ Office of Research and Development United States Environmental Protection Agency Duluth Minnesota USA; ^25^ Aquaculture Department SINTEF Ocean Trondheim Norway; ^26^ Smithsonian Tropical Research Institute Balboa, Ancon Republic of Panama; ^27^ School of Biological Sciences, Swire Institute of Marine Science The University of Hong Kong Hong Kong China; ^28^ Guangdong Basic Research Center of Excellence for Ecological Security and Green Development, Guangdong Provincial Key Laboratory of Water Quality Improvement and Ecological Restoration for Watersheds, School of Ecology, Environment and Resources Guangdong University of Technology Guangzhou China; ^29^ University of Trier, Biogeography Trier Germany; ^30^ Natural History Museum University of Oslo Oslo Norway; ^31^ Trace and Environmental DNA (TrEnD) Lab, School of Molecular and Life Sciences Curtin University Perth Australia; ^32^ Minderoo OceanOmics Centre at UWA, Oceans Institute University of Western Australia Crawley Australia; ^33^ Biology of Aquatic Population Laboratory Antananarivo University Antananarivo Madagascar; ^34^ Laboratoire de Biologie des Organismes et des Écosystèmes Aquatiques‐BOREA Muséum National d'Histoire Naturelle, SU, CNRS, IRD, UA Paris France; ^35^ School of Science, Engineering & Environment University of Salford, Newton Building, Crescent Salford UK; ^36^ Department of Biology University of Graz Graz Austria; ^37^ Department of Ecosystem Management, Climate and Biodiversity, Institute of Hydrobiology and Aquatic Ecosystem Management BOKU University Vienna Austria; ^38^ Animal Ecology Federal Institute of Hydrology Koblenz Germany; ^39^ CAS Key Laboratory of Marine Ecology and Environmental Sciences, Institute of Oceanology, Chinese Academy of Sciences Qingdao China; ^40^ School of Ecology and Environmental Sciences Yunnan University & Yunnan Key Laboratory for Plateau Mountain Ecology and Restoration of Degraded Environments Kunming China

**Keywords:** bioinformatics, environmental DNA, fish diversity, meta‐analysis, metabarcoding, river ecosystem

## Abstract

The analysis of environmental DNA (eDNA) has revolutionized biodiversity assessments in aquatic ecosystems, enabling non‐invasive monitoring of fish communities across diverse regions. However, the global comparability of these eDNA datasets remains ambiguous due to heterogeneous sampling protocols and bioinformatic workflows across studies, making it difficult to assess how robust and comparable the biodiversity patterns inferred from these datasets actually are. Here, we conducted a meta‐analysis of 58 riverine fish eDNA metabarcoding studies, covering 1818 sampling sites worldwide, to evaluate the robustness of eDNA‐derived biodiversity patterns. We found that species richness estimates and metrics of community structure derived under a common bioinformatic workflow were overall consistent with those of original analyses, despite the relatively high variability in bioinformatic analyses in the respective original studies. Contrastingly, congruence of species identity varied more extensively across datasets, mostly reflecting different completeness and regional relevance of reference databases. Restricting taxonomic assignment to basin‐specific species pools improved species identification accuracy, while datasets lacking publicly accessible or well‐curated reference data were more prone to mismatches. Year of sampling had a positive effect on taxonomic congruence, such that more recent studies showed increased robustness, also reflecting improved reference database coverage and enhanced species‐level identification over time and overall method congruence in more recent years. Overall, the suitability and potential of eDNA for global biodiversity monitoring is corroborating overall robust biodiversity estimates, irrespective of the bioinformatic approaches. Our study underlines the effectiveness and need for further harmonization of bioinformatic workflows and strengthened region‐specific reference databases for improved taxonomic resolution and comparability across studies.

## 1 Introduction

Freshwater ecosystems, particularly riverine systems, are important biodiversity hotspots, but face some of the highest rates of biodiversity loss (Almond et al. 2022; Hughes, Harrison, et al. 2021). Halting this decline is a central target in both the Global Sustainable Development Agenda and the Kunming‐Montreal Global Biodiversity Framework (CBD 2022; Obrecht et al. 2021; Tickner et al. 2020). Effective and sustainable monitoring of river biodiversity is a fundamental step in achieving these global biodiversity goals (Gonzalez et al. 2023). However, biodiversity assessments remain limited in many regions, particularly in the Global South, where freshwater biodiversity is often rich yet poorly studied (Chapman et al. 2024). Consequently, many regions and species remain underrepresented or unidentified in existing datasets, constraining conservation planning and policy development (Chapman et al. 2024; Senior et al. 2024). Therefore, rapid, cost‐effective, and standardized tools are urgently needed to assess biodiversity across riverine ecosystems worldwide.

Environmental DNA (eDNA) has emerged as a transformative tool for biodiversity monitoring, offering a noninvasive method to detect species across ecosystems, with particular effectiveness in riverine ecosystems (Altermatt et al. 2025; Blackman et al. 2024; Coutant et al. 2023; Yatsuyanagi et al. 2024). By capturing and analysing genetic material from environmental samples, eDNA enables species identification without the need for direct organismal observation (Pawlowski et al. 2020; Taberlet et al. 2012). The approach is particularly valuable for studying rare and cryptic species, such as fish, which are often hard to capture using conventional gillnet or electrofishing techniques (Cilleros et al. 2019; Piggott et al. 2021; Yao et al. 2022). eDNA also enables the integration of genetic signals across river networks, offering a more comprehensive view of biodiversity by capturing species distributions at large spatial scales (Carraro et al. 2020; Urycki et al. 2024).

While eDNA holds great promise for global biodiversity assessments (Altermatt et al. 2025), achieving comparability across existing datasets remains a significant challenge (Blackman et al. 2024; Loeza‐Quintana et al. 2020). Several aspects of sample collection and processing, including primer selection (Yang et al. 2023; Zhang et al. 2020), sample volume (Sakata et al. 2020; Zhang et al. 2023), sampling strategy (Zhang et al. 2023), and sequencing platforms (Korostin et al. 2020; Singer et al. 2019), have been shown to influence species detection and biodiversity estimates. However, less attention has been given to subsequent bioinformatics workflows and bioinformatic analysis of the data (but see Marques et al. (2020) and Mathon et al. (2021)). The specifics of bioinformatic workflow and analysis can significantly affect species identification and the consistency of results across studies (Mathieu et al. 2020). In other words, standardizing these workflows under common bioinformatic pipelines could improve the consistency and reliability of (re)analyses, enabling more reliable comparisons across studies. It is this long‐term value of eDNA metabarcoding results, namely that the sequences can be reanalyzed and used across studies, that gives the method a high applicability for global (meta)analyses and intercalibration and use of data. To effectively achieve comparability and robustness across studies, an understanding of the consistency and variability introduced by bioinformatic approaches and reference databases used is critical. Yet, variations in bioinformatic pipelines, including sequence quality filtering, clustering methods, and reference database quality, can lead to discrepancies in species identification (Keck, Couton, and Altermatt 2022; Marques et al. 2020), such that meaningful comparisons between studies are not a priori guaranteed. Specifically, data cleaning steps, including raw read quality control, clustering criteria, and abundance/occurrence thresholds, can impact the biodiversity estimates (Marques et al. 2020). The stringency of quality control affects the rates of false negatives, particularly for rare species that fall below detection thresholds, while less rigorous filtering can allow contaminant ASVs/OTUs to remain, leading to false positives that distort biodiversity estimates (Zinger et al. 2019). Further, the choice and quality/extent of the reference database used for matching sequences to taxonomic units is particularly important and profoundly affects species identification (Keck, Couton, and Altermatt 2022; Weigand et al. 2019). In summary, standardized bioinformatics workflows could play a crucial role in improving the reliability and comparability of eDNA‐based biodiversity assessments, depending on their effectiveness in minimizing variability across studies, and may enable a new, integrative view of biodiversity globally.

**FIGURE 1:**
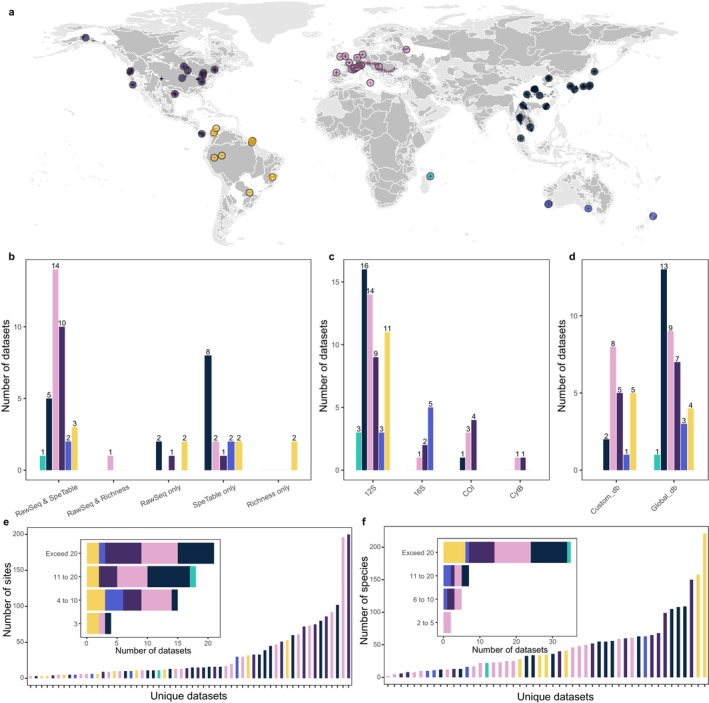
Distribution and summary of the 58 studies. Panel (a) shows the geographical distribution of the 58 studies (circles) and 1818 sampling sites (crosses). Light grey areas represent all global river basins, while dark grey areas indicate the river basins covered by the studies. Panels (b–d) summarize the available data types (raw sequencing data, species‐site table, and species richness), barcode region (12S, 16S, COI, or CytB), and the type of reference database used in the original species assignments (global or custom). Panel (e) summarizes the number of sites included in the datasets. Panel (f) provides a summary of the number of fish species in the originally classified datasets. The colours used in all panels correspond to the six continents.

Here, we tested the comparability and integration of eDNA studies by conducting a global meta‐analysis of 58 eDNA studies focused on riverine fish communities, integrating a broad set of published and unpublished eDNA datasets (Figure 1 and Figure S1). Specifically, we reanalyzed 56 original raw sequencing datasets using common bioinformatic pipelines, addressing three key questions: (1) How congruent are fish biodiversity patterns, focusing on both the species richness and community structure, when reanalyzing them in one common bioinformatic approach and comparing them to the original individual analyses? (2) To what extent do species identities align between reanalyzed and original results? (3) How much do the above comparisons depend on methodological choices? We aimed to disentangle the impacts of several factors that influenced the congruence between reanalyzed and original eDNA data sets, and particularly focused on effects of geographic context such as the continent where the study was conducted, methodological choices including the barcode or primer used and sampling years, as well as the completeness and type of reference database used in the original taxonomic assignment. By addressing these questions and the respective influence of these factors, we can improve our understanding of how eDNA‐based biodiversity assessments can be standardized and integrated and provide recommendations for enhancing the comparability and reliability of eDNA studies in general.

## 2 Method

### 2.1 Datasets Compilation

A comprehensive literature review was conducted on December 13, 2022, using the Web of Science Core Collection, with the full search query provided in the Supporting Information Text. The initial search returned 673 records, which were first filtered by removing 13 book chapters, leaving 660 records. These records were sorted by relevance and manually reviewed for inclusion based on the following criteria: (1) literature type—review and meta‐analysis papers were excluded; (2) target taxonomic group—only studies focused on fish communities were considered; (3) ecosystem—only studies on rivers or streams were included; (4) biomonitoring method—only studies using eDNA metabarcoding were kept.

After this initial screening, 50 relevant papers were selected. These papers were further assessed for data availability, with the following criteria for inclusion: (1) studies that provided geographic coordinates for sampling sites; (2) studies that reported species richness or provided raw sequencing data. This second review led to the retention of 42 papers. From these, metadata were extracted, including publication details, data availability, sampling protocols, metabarcoding procedures, and bioinformatic methods (Table S1).

To expand our dataset and capture additional relevant studies, we made a public call on social media platform X (back then known as Twitter), targeting researchers and institutions involved in similar studies. This outreach aimed to gather unpublished or less accessible data to enhance the comprehensiveness of our datasets. As a result, 23 further individual studies were provided by co‐authors, contributing data from 588 sampling sites across 19 river catchments. Of the studies provided, one was from Africa, five from Asia, two from Australia, seven from Europe, five from North America, and three from South America. Three of the studies had already been included during the paper review and screening process, bringing the total to 62 studies (see “DataID” in Table S1). In Table S1, metadata on all of these studies were provided, covering details on sampling (e.g., time, frequency, number of sites, volume), metabarcoding experiments (e.g., DNA extraction, PCR, sequencing), and bioinformatics (e.g., quality control, clustering, taxonomy assignment) aspects. For each study, raw sequencing data, species richness estimates for each sampling site (species richness table), and species‐site matrixes were extracted. Four studies, each with only a single sampling site, were excluded, resulting in a final set of 58 studies used in the meta‐analysis (Figure 1 and Figure S1).

Among them, 41 studies contained raw sequencing data, with some using multiple primers (Figure 1). To account for this, the studies were further separated based on unique study by primer combinations, resulting in a total of 56 distinct raw sequencing datasets (i.e., study by primer combinations; referred as “dataset” hereafter), each archived in separate folders (see “RawSeqID” in Table S1). Primer names were standardized based on their sequences, and sample names were checked and standardized to align with the species‐site matrixes or species richness tables. All fish species names were standardized using “rfishbase” R package (Froese and Pauly 2002).

We further classified the datasets into two groups based on whether a global or custom reference database was used in the original assignments (Figure S1). The global reference database included sources such as GenBank, ENA, MitoFish. The custom reference databases were defined as: (1) using barcodes from local species sampled within the region, (2) curating a reference database from the global reference database by retaining only barcodes of historically recorded species, and (3) assigning species against the global reference database and then correcting based on local historical records.

To assess the methodological and geographical representativeness of reanalyzable datasets (with raw sequencing data), we first compared reanalyzable and non‐reanalyzable datasets in terms of sampling year and volume using Wilcoxon rank‐sum tests for continuous variables (Fix and Hodges Jr 1955). Next, we applied Fisher's exact tests to compare the distribution differences of geographical region (continent) and barcode used between the reanalyzable and non‐reanalyzable datasets (Upton 1992).

### 2.2 Reference Database Curation

The CRUX pipeline, the first module of the Anacapa Toolkit (Curd et al. 2019), was used to curate reference databases for specific primer sets. Briefly, the process begins with in silico PCR using ecoPCR on the EMBL nucleotide sequence database to generate a seed library of taxon‐specific identifiers (Ficetola et al. 2010; Stoesser et al. 2002). The pipeline then verifies the amplicon match by checking primer regions and trimming them using CUTADAPT (Martin 2011). Since many GenBank sequences do not include primer regions, CRUX performs two rounds of BLAST against the NCBI nucleotide database (Camacho et al. 2009). The first round filters for full‐length reads, while the second includes shorter reads (down to 70% of the full length). The results are de‐replicated, retaining only the longest reads. CRUX also generates a taxonomy file using entrez‐qiime, mapping reads to taxonomic categories (Baker 2016). Reads with incomplete or unclear taxonomy (e.g., “unassigned”, “uncultured”) are excluded to maintain high‐quality reference data. The parameters for each primer set are outlined in Table S2. Finally, the reference databases are formatted into Bowtie2‐formatted index libraries.

### 2.3 Bioinformatics Workflow

For datasets that were not demultiplexed, paired‐end reads were first merged using VSEARCH (Rognes et al. 2016). The *barcode_splitter* was used for demultiplexing raw sequencing reads based on barcode sequences, allowing up to three mismatches (Leach and Parsons 2019). Any reads that did not match a barcode or had multiple barcode matches were discarded. Demultiplexed reads were renamed and organized into separate fastq files for each sample. Additionally, *NGSFILTER* from the OBITools package was used for filtering and processing in cases where double‐barcode adapters were used (Boyer et al. 2016).

The DADA2 pipeline was then run in the R environment to process the demultiplexed sequencing reads (Callahan et al. 2016). Initially, reads were imported and filtered to remove ambiguous bases using the *filterAndTrim()* function, with a maximum of 0 ambiguous bases. Primer sequences were identified and removed using CUTADAPT, with primer sequences checked in all orientations to ensure accurate trimming (Martin 2011). Low‐quality reads were discarded using the *filterAndTrim()* function, with a maximum expected error rate (maxEE) of 2 and a truncation quality (truncQ) threshold of 2. For each demultiplexed sample, error rates were learned using *learnErrors()*, and reads were denoised with the *dada()* function to generate amplicon sequence variants (ASVs). Paired‐end reads were merged using *mergePairs()*, and chimeric sequences were removed using *removeBimeraDenovo()*. A sequence table was created, retaining only chimera‐free ASVs. The number of reads at each step of processing (raw, filtered, denoised, merged, and non‐chimeric) was tracked for each sample.

The taxonomy of the ASVs was assigned using the Classifier module of the Anacapa Toolkit, which employs *Bowtie2* and a Bayesian Least Common Ancestor (BLCA) algorithm (Curd et al. 2019; Gao et al. 2017; Langmead and Salzberg 2012). Initially, all ASVs were globally aligned to the CRUX database using Bowtie 2. The top 100 hits were then processed using the BLCA script for taxonomic assignment. The confidence of each taxonomic assignment was assessed using bootstrap scores. The species annotations in the output were initially uncorrected based on the local species and were therefore referred to as “global assignments” throughout the manuscript.

### 2.4 Local Species Record and Reference Database Coverage

We used a global database of freshwater fish occurrence, compiled through an extensive literature review and online resources, which provides complete species lists, including well‐known native, introduced, and invasive fishes, for 3119 drainage basins (Tedesco et al. 2017). This database was used to define the local species list for each dataset, identified at three hierarchical scales: basin, country, and continent. First, each dataset was assigned to a specific drainage basin. For datasets not directly linked to a basin in the database, the nearest available basin was used as a proxy. Fish records for the assigned basin, country, or continent, respectively, were then retrieved to compile the local species list for each dataset.

We quantified the coverage of the local species list by the barcode reference database. For each dataset, we used the barcode reference database corresponding to the barcode region targeted by the primers used in that dataset (generated in section “Reference database curation”). Coverage was defined as the proportion of taxa in the local species list that were represented in the barcode reference database. This was calculated at both species and genus resolution and for each geographic scale.

Separately from coverage, we compared the global assignment results (generated in section “Bioinformatics Workflow”) with the local species list. For each dataset, geographic scale, and taxonomic resolution (species or genus), we summarized the number of taxa in the global assignment results that were present in the corresponding local species list.

To ensure consistency, the fish species names in the global assignments results, barcode reference database, and local species lists were validated and standardized using the “rfishbase” R package (Froese and Pauly 2002). Differences between continents and barcodes in (i) coverage of the local species list by the barcode reference database and (ii) the number of assigned taxa of the global assignment results present in the local species lists were assessed by the Kruskal‐Wallis rank sum test (Ostertagova et al. 2014) followed by post hoc Dunn's test after checking the normality and homogeneity of variances (Table S3). The *multcompLetters2()* function from the “multcompView” R package was used to extract group‐wise significance letters based on *p* values from Dunn's test.

To validate the local species lists and determine the appropriate geographical scale and taxonomic resolution for subsequent analyses, we re‐examined the comparison between the global assignment results and the local species lists for datasets from Switzerland (Europe), French Guiana (South America), the St. Regis River (North America), and the Chao Phraya River (Thailand), where historical fish records are publicly available (Table S4).

### 2.5 Comparison of Species Richness Estimates and Community Structures

We compared species richness estimates between the original and reanalyzed assignments using generalized linear mixed models (GLMM) with a negative binomial error distribution and a log link, with dataset identity included as a random factor (Brooks et al. 2017). Model adequacy was assessed using simulation‐based residual diagnostics, including tests for overdispersion and zero inflation, implemented via the “DHARMa” package in R (Hartig and Hartig 2017). For the reanalyzed assignments, we considered four types: (a) global assignments (uncorrected using local fish lists), (b) assignments corrected to the basin‐scale local species, (c) assignments corrected to the country‐scale local species, and (d) assignments corrected to the continent‐scale local species.

To further assess the robustness of community structures across continents and assignment types, we extracted the full species list for each dataset and each assignment type (original assignment, re‐assignments at the global level, and re‐assignments at the basin level), and combined these into a single species occurrence matrix. We then calculated the Jaccard distance and performed Non‐metric Multidimensional Scaling (NMDS), followed by PERMANOVA and ANOSIM tests using the “vegan” R package to evaluate the differences in community composition (Dixon 2003). To account for potential differences in multivariate dispersion across groups, we performed the Multivariate Dispersion Analysis (*betadisper()*), which tests for homogeneity of multivariate dispersion among groups (Anderson 2006). This was followed by db‐RDA (distance‐based redundancy analysis) to examine the differences in community composition while considering the multivariate dispersion (Legendre and Anderson 1999). Both Multivariate Dispersion and db‐RDA were performed using the “vegan” R package.

### 2.6 Comparison of Species Identity

The species identities were compared between the original assignments and the reanalyzed assignments corrected to the basin‐level local species. All species were classified into four categories: (1) shared species, (2) species unique to the re‐assignments, (3) species unique to the original assignments but considered local species at the basin level, and (4) species unique to the original assignments but not local species at the basin level. The proportions of shared species in the re‐assignments and original assignments (proportional data), as well as the number of additional local species found in the original assignments (count data), were assessed for normality using the Shapiro–Wilk test and for homogeneity of variance using Levene's test. The results indicated that the data violated assumptions of normality and homogeneity of variance (Table S3), so instead of ANOVA, comparisons were made between continents using the Kruskal‐Wallis rank sum test followed by post hoc Dunn's test (Ostertagova et al. 2014). The differences between datasets using either the global or custom reference database were assessed by the Wilcoxon test (Fix and Hodges Jr 1955).

To evaluate whether methodological factors influence the agreement between original and reanalyzed taxonomic assignments, we conducted two complementary analyses: (i) dataset‐level overlap analysis and (ii) species‐level relative abundance analysis. First, generalized linear models (GLMs) were used to examine whether methodological factors influence the agreement between original and reanalyzed assignments at the dataset level (Hastie and Pregibon 2017). Agreement was quantified using two complementary overlap metrics that were analysed in separate models. The first response variable was the proportion of shared species, calculated as

$$p_{\mathrm{sp}}=\frac{N_{\text{shared species}}}{N_{\text{species in reanalysis}}}$$

The second response variable was the proportion of sequencing reads assigned to shared species, calculated as follows:

$$p_{\text{read}}=\frac{N_{\text{reads assigned to shared species}}}{N_{\text{total assigned reads in reanalysis}}}$$

Because each dataset contributes a single observation for each response variable, dataset identity corresponds to the observational unit and no within‐dataset replication exists. Therefore, no “study identity” random effects were included in these dataset‐level models. Both response variables were modelled using a beta error distribution with a logit link. We used the number of reads assigned to fish as the predictor variable, rather than raw sequencing depth, because a significant portion of reads were annotated as other vertebrates, making fish reads more relevant for determining the proportion of shared species across datasets (Figure S2).

Second, to compare relative abundance patterns at the species level, reanalyzed species within each dataset were classified as either shared with the original assignment or unique to the reanalysis. For each species, we quantified relative read abundance using two complementary indices. The first index measured the proportion of total reads across all sites within a dataset that were assigned to that species, calculated as follows:

$$a_{i,d}=\frac{\sum_{s\in d}R_{i,s}}{\sum_{s\in d}\sum_{j}R_{j,s}}$$

where $R_{i,s}$ is the read count of species *i* at site *s* and *d* denotes the dataset. The second index measured the mean within‐site relative abundance across sites, calculated as

$$b_{i,d}=\frac{1}{n_{d}}\sum_{s\in d}\frac{R_{i,s}}{\sum_{j}R_{j,s}}$$

where $n_{d}$ is the number of sites in dataset *d*. We tested whether both indices differed between shared and unique species and whether any differences were associated with methodological factors. Because multiple species were observed within each dataset, dataset identity was included as a random intercept in mixed‐effects models to account for non‐independence (Brooks et al. 2017).

## 3 Results

### 3.1 Summary of the Datasets

A total of 58 studies (see “DataID” in Table S1), encompassing 1818 sampling sites globally, were included in the meta‐analysis (Figure 1). These studies were distributed across different continents, with 15 from Asia, 17 from Europe, 12 from North America, 9 from South America, 4 from Oceania, and 1 from Africa. Among these, 11 studies utilized multiple barcode markers. The majority (51 of 58) employed the 12S rRNA gene, followed by 6 using 16S rRNA, 7 using COI (Cytochrome C Oxidase subunit I gene), and 2 using CytB (Mitochondrially Encoded Cytochrome B gene). Regarding species assignment, 21 studies used custom reference databases, while the remaining studies relied on global reference databases, which did not account for historical species occurrences. The number of sampling sites per dataset ranged from 3 to 200, with 19 studies containing fewer than 10 sites, 18 covering 11 to 20 sites, and 21 including more than 20 sites. The number of species detected per dataset ranged from 2 to 221, with 35 studies identifying more than 20 species.

Of the 41 studies with available raw sequencing data (Figure 1), seven employed more than one primer set. After organizing the raw sequencing data by dataset identity and primer used, we identified a total of 56 distinct raw sequencing datasets (i.e., unqiue study by primer combinations; see “RawSeqID” in Table S1). Six of these lacked sequencing indexes required to distinguish individual samples, so finally 50 raw sequence datasets were reanalyzed using a standardized bioinformatics workflow.

For the methodological and geographical representativeness of reanalyzable datasets (with raw sequencing data), we found no significant differences between reanalyzable and non‐reanalyzable datasets for sampling year (Figure S3, Wilcoxon *p* = 0.580), sampling volume (Wilcoxon *p* = 0.387), or barcode region used (Fisher's exact test *p* = 0.362). However, we observed a significant geographical disparity, with a higher proportion of reanalyzable datasets from regions like North America (Fisher's exact test *p* < 0.001).

### 3.2 Reference Database Coverage of the Local Species

Out of the 50 raw sequencing datasets, 23 distinct primer sets were used, and reference databases were curated separately for each primer set. These primer sets covered a broad spectrum of species according to the in silico results, with varying amplification capabilities against GenBank (Table S5). Approximately 4000 species had 12S rRNA barcodes corresponding to 11 primer sets, amplifying between 3785 and 4387 species—except for the Riaz02 primer set, which could only amplify 4 species. Five primer sets targeting 16S rRNA genes successfully amplified between 4166 and 9022 fish species, while five COI primer sets amplified between 5307 and 9761 species. In contrast, the CytB primer sets were more limited, amplifying 2571 species with Cytb01 and 1705 species with Cytb02.

**FIGURE 2:**
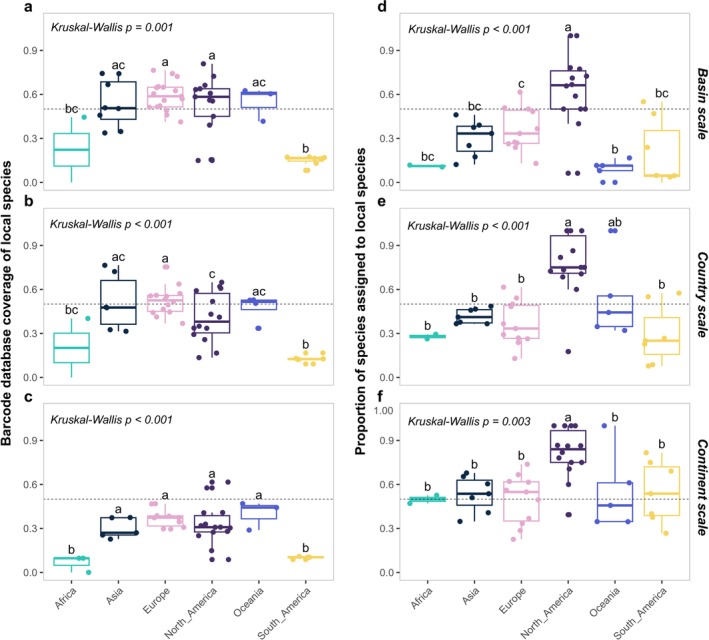
Barcode reference database coverage of the local species list and the proportion of species assigned as local taxa using global assignments across different spatial scales for each dataset. Local species were defined at the basin (a, d), country (b, e), and continent (c, f) scales, respectively. Panels (a–c) show coverage, defined as the proportion of species in the local species list that were represented in the barcode reference database. Panels (d–f) show the proportion of species in the global assignment results that were present in the corresponding local species list. The differences between continents were assessed by ANOVA and subsequent pairwise Dunn's tests. Different letters indicate statistically significant differences between groups (*p* < 0.05). Groups sharing the same letter are not significantly different, whereas groups with no letters in common differ significantly. The horizontal dotted lines indicate a value of 0.5.

The coverage of the local species list by the barcode reference database was assessed across datasets at different geographical scales (basin, country, or continent), revealing substantial variability based on both the barcode used and the continent of the datasets. At the species level, coverage of the local species list by the barcode reference database varied widely across datasets and continents (Figure 2a–c), with consistently lower coverage in Africa and South America compared with Asia, Europe, and North America. Comparisons of barcode coverage by different barcode regions showed significant differences at the species level at both the basin (Kruskall‐Wallis *H* = 17.75, *p* = 0.001) and continent (Kruskall‐Wallis *H* = 20.23, *p* < 0.001) scales (Figure S4). The datasets using 12S rRNA genes generally exhibited a wide range of coverage of the local species list by the barcode reference database, with consistent patterns across geographical scales (Figures S4 and S5). Genus‐level coverage results were largely congruent with the species‐level patterns and are provided in the Supporting Information (Figures S6 and S4).

Separately, the global assignments were compared with local species lists at different geographical scales, revealing significant differences between datasets from various continents (Figure 2d–f), while there were no significant differences in the proportion of species assigned as local between different barcodes (Figure S7). At the basin scale, the mean proportion of species assigned as local was 63.3% across datasets, and proportions generally increased when local species were defined at broader (country and continent) scales. Datasets from North America showed the highest proportion of local species, but they were markedly lower in Oceania, Africa, and South America. In contrast, datasets from Asia and Europe showed lower local species assignment at the basin scale (30.1% for Asia and 36.2% for Europe), with higher proportions at the continent scale (53.4% for Asia and 49.6% for Europe). Genus‐level assignment trends were largely congruent with species‐level results and were therefore reported in the Supporting Information (Figures S6 and S7).

### 3.3 Consistency in Species Richness Estimates and Community Structure

The reanalyzed species richness estimates showed clear patterns across datasets but varied significantly depending on the spatial scales used to define local species. The original assignments identified a total of 1521 species, with an average of 17 species per site (ranging from 0 to 106), while the reanalyzed global assignments identified 1544 species, averaging 20 species per site (ranging from 0 to 337). When restricted to local species, the total species richness was reduced, with 895 species at the continent scale (average 13 species per site, ranging from 0 to 190), 762 species at the country scale (average 11 species per site, ranging from 0 to 181), and 661 species at the basin scale (average 10 species per site, ranging from 0 to 149).

**FIGURE 3:**
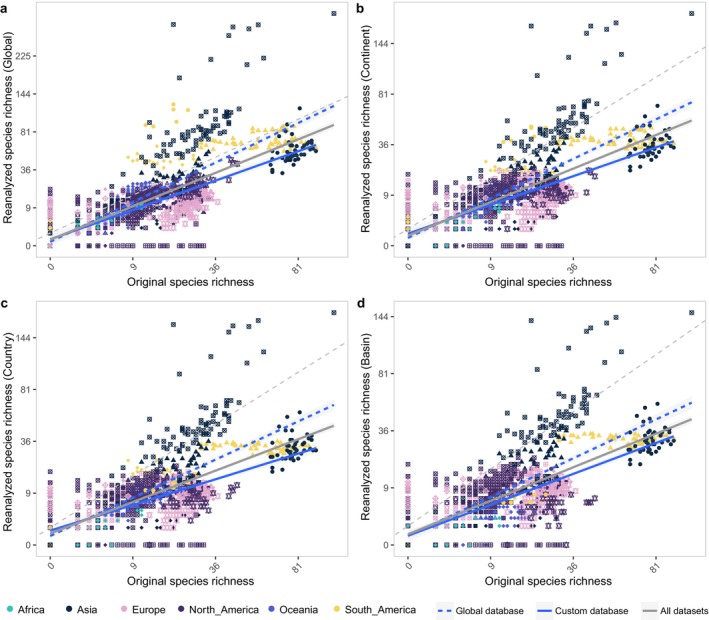
Comparison of species richness estimates between original and reanalyzed assignments. Panel (a) shows species richness based on global species assignments. Panels (b) to (d) display species richness estimates reanalyzed by restricting to local species at the continent (b), country (c), and basin (d) scales, respectively. The grey dashed line represents 1:1 line. All regressions were performed using generalized linear mixed models (GLMM), with dataset identity as a random factor to account for variation between datasets. The grey solid lines represent the regression for all datasets, the blue solid lines correspond to datasets that originally used custom reference databases for species assignment, and the dashed blue lines represent datasets originally using global reference databases. Point shapes corresponded to different dataset IDs for which raw sequence data were available.

The GLMM results revealed overall consistent patterns between reanalyzed and original species richness estimates across all datasets and geographical scales (Figure 3, Table S6). For all datasets, original species richness had a positive but relatively weak effect on the variation in reanalyzed species richness (e.g., *R* ^2^_Marginal_ = 0.04, *R* ^2^_Conditional_ = 0.97, *t* = 15.61, *p* < 0.001 at the global scale), indicating that most explanatory power came from the random effect of dataset ID, which captured dataset‐specific variability. When datasets were split based on their use of global or custom reference databases in the original assignments, those with a global reference database showed stronger consistency between the reanalyzed and original estimates. At the global scale, the GLMM results for the datasets using global reference databases showed higher performance (*R* ^2^_Marginal_ = 0.18, *R* ^2^_Conditional_ = 0.94, *t* = 16.26, *p* < 0.001), with similar consistency at continent (*R* ^2^_Marginal_ = 0.16, *R* ^2^_Conditional_ = 0.93, *t* = 15.06, *p* < 0.001), country (*R* ^2^_Marginal_ = 0.16, *R* ^2^_Conditional_ = 0.92, *t* = 15.17, *p* < 0.001), and basin scales (*R* ^2^_Marginal_ = 0.13, *R* ^2^_Conditional_ = 0.92, *t* = 13.80, *p* < 0.001). In contrast, datasets using custom reference databases in their original assignments showed weaker consistency, with *t*‐values consistently below 10 and marginal 𝑅^2^ values explaining less than 1% of the variance (yet the conditional *R* ^2^ above 0.90) at all scales.

**FIGURE 4:**
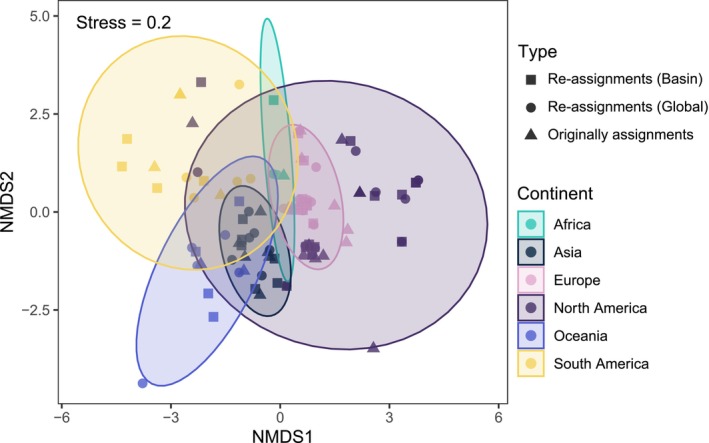
NMDS plots showing community composition across datasets. The colours represent different continents, and the shapes indicate the dataset type: Original assignment, global re‐assignments, and basin‐scale re‐assignments.

Overall community composition remained consistently robust across species assignment types and was primarily structured by continents (Figure 4, Table S7). PERMANOVA results revealed a significant effect of continents on community composition (*R* ^2^ = 0.19, *p* = 0.001), while species assignment types did not have a significant impact (*R* ^2^ = 0.019, *p* = 0.137). Similarly, ANOSIM confirmed a significant difference in community composition between continents (*R* = 0.57, *p* = 0.001) but found no significant differences between species assignment types (*R* = 0.0031, *p* = 0.346). Multivariate dispersion analysis revealed a significant difference in the multivariate dispersion between continents (*F* = 5.84, *p* = 0.001), indicating unequal dispersion among groups. db‐RDA confirmed significant centroid differences between continents (*F* = 5.28, *p* = 0.001), further supporting the results from PERMANOVA and ANOSIM.

### 3.4 Comparison of Species Identity Across eDNA Studies

**FIGURE 5:**
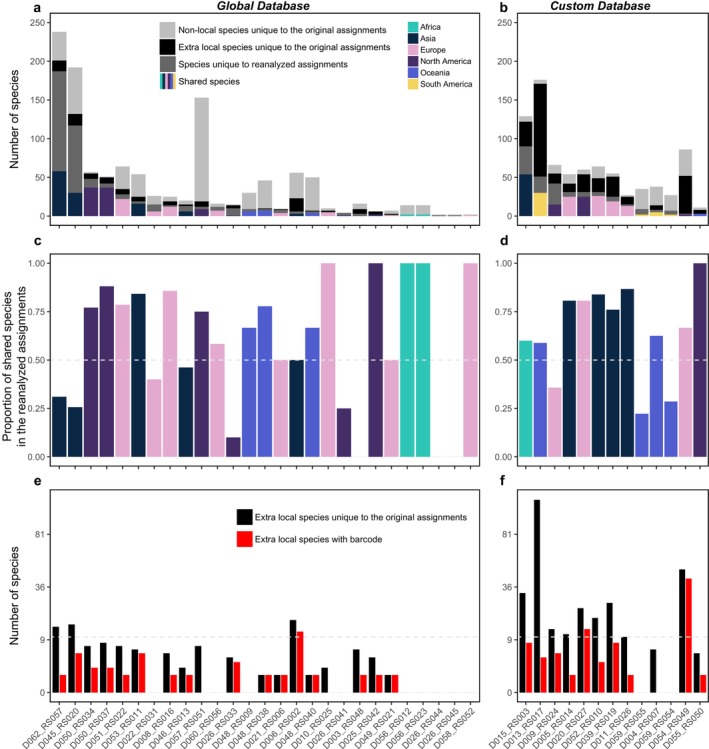
Comparison of species identity between the original and reanalyzed assignments for each dataset. The reanalyzed assignments only include species historically recorded at the basin scale. Panels (a–b) show the overlap between the original and reanalyzed assignments, with shared species colour‐coded by continent. Dark grey bars represent species unique to the reanalyzed assignments, black bars indicate species unique to the original assignments but historically recorded at the basin scale (extra local species), and light grey bars represent species unique to the original assignments but not historically recorded. Panels (c–d) show the percentages of species detected in the reanalyzed dataset that were also found in the original dataset. Panels (e–f) show the number of extra local species in the original assignments, along with the number of these species with barcode records in GenBank. The x‐axis in all panels represents individual dataset IDs. Assignments were classified based on the original assignment using either a global reference database (a, c, e) or a custom reference database (b, d, f).

Out of the 50 datasets with raw sequencing data available reanalyzed, 46 were successfully annotated to basin‐level local fish species. Among these 46 datasets, 40 had original species assignment tables available, enabling a direct comparison between the original and reanalyzed species identities (Table S1). The species shared between the original and reanalyzed assignments ranged from 0 to 58 species per dataset (Figure 5 and Figure S8), corresponding to 0%–100% of species counts (mean: 60.71%, median: 66.67%). These shared species accounted for 0% to 100% of sequencing reads, with a mean and median overlap of 65.3% and 82.7%, respectively (Figure S9). Significant differences were observed between continents for the percentage of shared species in the reanalyzed assignments (Table S8, Kruskall‐Wallis *H* = 12.14 *p* = 0.033), while no significant differences were found between datasets originally analysed using global versus custom reference databases (Table S9, Wilcoxon *p* = 0.734).

Despite the overlap, some species were unique to the original assignments as basin‐scale local species (extra local species hereafter), with 11 species per dataset on average, ranging from 0 to 120 (Figure 5, Figure S8). No significant differences in the number of extra local species were found between continents (Table S8, Kruskall‐Wallis *H* = 15.94 *p* = 0.159), but datasets using custom reference databases in the original assignments had significantly more extra local species (range from 0 to 120) compared to those using global reference databases (Table S9, range from 0 to 17; Wilcoxon *p* = 0.003). In general, only 0 to 13 of these extra local species appeared in the curated reference database used for reanalysis. An exception was dataset D054, which had the lowest reads per sample (2597 reads, Table S10), resulting in 4 out of 443 ASVs being identified as fish (Figure S10). In contrast, other datasets had an average of 9943 to 3,704,035 reads per sample (Table S10).

**FIGURE 6:**
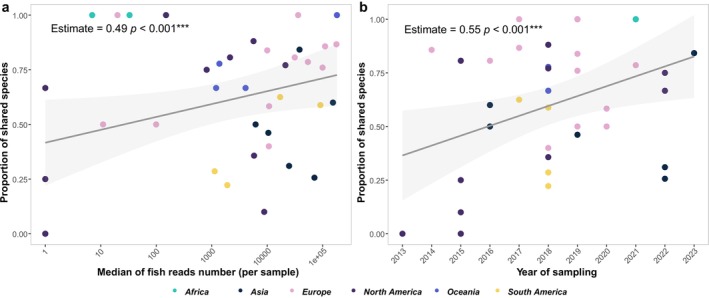
Impacts of fish reads number per sample and sampling year on the proportions of shared species between reanalyzed and original assignments. Panels (a) and (b) show the effects of the median sequencing depth of datasets (a) and the year of sampling (b), respectively. In both panels, the color of the data points represents different continents. The regression lines, based on a generalized linear model, illustrate the trends, with the shaded areas representing 95% confidence intervals.

The modelling results revealed that discrepancies between original and reanalyzed assignments were largely driven by the number of reads assigned to fish per sample, sampling year, and species richness, whereas the reference database type had minimal impact (Figure 6 and Table S11). The proportion of shared species (based on species count) increased significantly with higher median fish reads number (*z* = 3.77, *p* < 0.001) and more recent year of sampling (*z* = 7.11, *p* < 0.001), but decreased with greater reanalyzed species richness (*z* = −4.67, *p* < 0.001). In contrast, the proportion of reads assigned to shared species was significantly affected only by the year of sampling, showing a higher overlap in more recent years (Figure S11 and Table S11, *z* = 5.53, *p* < 0.001). Reference database type (global vs. custom) had no significant effect in either model. Species unique to the reanalyzed datasets had significantly lower total relative abundance (*z* = −3.01, *p* = 0.003) and lower mean relative abundance across sampling sites (*z* = −3.41, *p* = 0.001) compared to shared species (Figure S12 and Table S12). Reference database type showed marginal effects on total (*z* = 1.84, *p* = 0.066) and mean (*z* = 1.99, *p* = 0.047) relative abundance, whereas the year of sampling had no significant effect.

To assess the potential influence of low median fish reads on the observed patterns, we conducted an additional sensitivity analysis by removing data points/dataset with low read numbers assigned to fish (5 studies removed; see Table S11). After removing these points, median fish reads number no longer had a significant effect on the proportion of shared species. This was to some level expected, as we removed the extreme values, indicating that datasets with a low number of fish reads were driving this relationship in our initial analysis. However, sampling year remained a significant factor influencing the proportion of shared species, reinforcing the conclusion that sampling year is a key factor in explaining the variation in shared species, independent of the number of fish reads per dataset.

## 4 Discussion

Environmental DNA has become a powerful tool for monitoring riverine fish biodiversity, providing a non‐invasive approach for large‐scale assessments (Blackman et al. 2024). However, questions remain about its global applicability, particularly the consistency of existing datasets generated through varying field and laboratory protocols. While a range of upstream factors, such as sampling volume and primer choice, have been addressed in previous studies, the influence of the bioinformatic workflow on species detections has been comparatively less discussed. The storage and availability of raw sequence data have been critical in enabling this reanalysis, and our study stands as one of the first and largest efforts to reanalyze fish eDNA datasets under a common bioinformatic framework. Specifically, our major reanalysis revealed that overall biodiversity patterns in terms of species richness and community structure were consistent, yet discrepancies did arise in the species identities between the reanalyzed and original assignments. These differences were not necessarily due to variations in the bioinformatic pipeline but rather stemmed from the choice and quality of reference databases. These findings underscore the potential of eDNA as a reliable and scalable tool for monitoring global fish biodiversity, while highlighting the importance of refining bioinformatic workflows and reference database curation for more accurate species identification.

As a key finding of our study, fish eDNA datasets were robust and revealed general biodiversity trends despite the inherent heterogeneity of studies and datasets, underscoring the potential of leveraging existing fish eDNA datasets for global biodiversity analyses. As eDNA metabarcoding continues to advance in biodiversity monitoring, concerns linger about how methodological factors, including bioinformatic approaches, may influence local diversity estimates (Blackman et al. 2024; Marques et al. 2020). Various bioinformatic tools, such as MiFish pipeline (Sato et al. 2018), OBITools (Boyer et al. 2016), Anacapa (Curd et al. 2019), and QIIME (Bolyen et al. 2019; Caporaso et al. 2010), offer comprehensive eDNA analysis workflows with the same primary objectives—inferring species identities and estimating species richness—but they employ different strategies (Hakimzadeh et al. 2024; Mathon et al. 2021). These workflows diverge in several critical areas, including OTU/ASV clustering methods, taxonomic annotation strategies, and criteria for defining taxonomic resolution (Hakimzadeh et al. 2024; Mathon et al. 2021). Moreover, the choice of reference databases is crucial for eDNA metabarcoding analysis (Keck and Altermatt 2023; Keck, Couton, and Altermatt 2022). Public reference databases offer greater taxonomic breadth but tend to have lower resolution for shorter eDNA barcodes and higher rates of errors/taxonomic misidentifications, which can result in misidentifications or false positives (Keck, Couton, and Altermatt 2022). On the other hand, local reference databases, though narrower in scope, tend to provide more accurate species identification due to their focus on regional taxa. Given these differences, the impact of bioinformatic workflows on the accuracy and consistency of biodiversity assessments is one of the central concerns when compiling eDNA datasets for global biodiversity insights (Blackman et al. 2024; Hakimzadeh et al. 2024; Mathon et al. 2021). Our reanalysis revealed the general consistency of species richness estimates and the robustness of community structures across studies employing a unified bioinformatic workflow (Figures 3 and 4), indicating existing eDNA data can offer reliable community‐level insights.

The choice and quality of reference databases critically influenced the accuracy of species identification in eDNA studies, with ongoing improvements over time helping to reduce taxonomic mismatches (Keck, Couton, and Altermatt 2022). In our reanalysis, species identity mismatches between original and reanalyzed assignments varied across datasets, largely reflecting differences in how reference databases were constructed and applied (Figure 5). First, our reanalysis revealed that restricting species identification to basin‐scale local species pools substantially improved taxonomic resolution, emphasizing the value of regionally tailored reference databases for accurate biodiversity assessments. For instance, datasets originally analysed using global reference databases often showed improved species annotations when reanalyzed with basin‐specific species lists, underscoring the importance of aligning reference data with local faunas. Conversely, in regions such as South America, where custom reference databases were used but not publicly archived (e.g., Cantera et al. (2022)), reanalysis suffered from lower taxonomic resolution due to limited access to these localized resources. Second, persistent gaps and geographic biases in reference database coverage remained a significant challenge. Previous studies have highlighted that gaps in reference database coverage for fish species were especially prominent in tropical regions, where both species diversity and the number of threatened species were highest (Marques et al. 2021). In Europe, while freshwater fish species were relatively well represented with 88% coverage of COI gene, the coverage for other markers such as 12S rRNA remains low, with only 36% of species covered (Weigand et al. 2019). Similarly, for Chinese fish species, around 60% were represented in reference databases, but nearly 90% of the sequences came from outside China, highlighting a critical geographical bias (Li et al. 2022). Our study found the uneven coverage of reference databases across continents, with notable gaps in the taxonomic representation of local fish species in South America and Africa, was the main reason for the species identities differences between original and reanalyzed results (Figure 2). Furthermore, our use of basin‐, country‐, and ecoregion‐level species pools, respectively, as a filter assumes that these occurrence lists are reasonably complete. In under‐surveyed regions, particularly in the tropics, incomplete or outdated checklists may lead us to discard genuine detections and thus slightly inflate apparent congruence between the original and reanalyzed assignments. Third, our results showed that the year of sampling was positively associated with species identification accuracy, indicating that improvements in reference databases over time could enhance biodiversity estimates (). Reanalysis of “old” datasets with updated reference libraries thus provides a valuable opportunity to improve consistency and comparability across studies. Overall, our findings highlighted the need for continued expansion and curation of region‐specific reference databases to support reliable and standardized eDNA‐based biodiversity monitoring.

While eDNA data provides a promising tool for global biodiversity assessments, the growing issue of spatial bias in eDNA research raises concerns. In the context of global biodiversity targets, such data discrepancies could lead to the systematic underestimation of biodiversity in underrepresented regions, thereby introducing bias into global biodiversity evaluations and subsequent scientific decision‐making (Chapman et al. 2024). Traditional biodiversity monitoring data, as represented by the Global Biodiversity Information Facility (GBIF), has already demonstrated geographic bias, with most data originating from high‐income regions (Chapman et al. 2024; Hughes, Orr, et al. 2021). Therefore, in addition to the need for more biodiversity data, there is an urgent need to focus on underrepresented areas and taxa. Our meta‐analysis shows that, aside from the geographic bias in the reference databases mentioned earlier, the geographic distribution of eDNA datasets also exhibits spatial bias, with a concentration of studies in Europe, North America, and East Asia, while regions such as South America, Africa, Oceania, and other parts of Asia remain under‐sampled (Figure 1). Similar to the study by Keck, Blackman, et al. (2022), eDNA metabarcoding studies, though covering multiple continents and climatic regions, still show a marked spatial clustering, with most studies focused on Europe and North America. This highlights the critical need for future eDNA research to address these geographic disparities by increasing efforts in underrepresented regions. Doing so will be essential to support a more comprehensive, equitable, and accurate global biodiversity conservation agenda.

Our reanalysis underscored the critical importance of raw data availability and comprehensive metadata in future eDNA studies. Not all datasets could be effectively used in the reanalysis. Out of the 42 published studies examined, raw sequence data were missing in 17 cases, while an additional four studies included raw sequencing data but lacked barcodes/indexes necessary to distinguish individual samples from sequencing files. Specifically, we observed a significant geographical disparity in the willingness to share raw sequencing data, with a higher proportion of reanalyzable datasets from regions like North America (Figure S3, Fisher's exact test *p* < 0.001), indicating that the coverage of our social‐media‐sourced studies depended on our own networks, geographic differences in social media use, and authors' willingness to share data, which may also be culturally influenced. As with many global ecological studies, this results in geographic differences in coverage and underscores the need for more systematic and open global data sharing in freshwater eDNA metabarcoding. Moreover, the absence of key metadata in some datasets hindered the reanalysis process. For example, some studies employed modified primer sets but failed to report the exact primer sequences, instead referencing the original publication. Other issues included inconsistencies between sequencing file names and specific samples, as well as the omission of sampling site coordinates. This highlights a critical issue: for eDNA data to be truly useful, careful management and appropriate data storage protocols are essential (Altermatt et al. 2025). In this study, we outlined key metadata including sampling, metabarcoding experiment process and detailed bioinformatics pipeline that should be reported to facilitate and enhance future reanalysis (Table S1). Looking ahead, it is crucial to establish standardized data storage protocols and ensure the reporting of comprehensive metadata for reanalysis (see Klymus et al. (2024) and Takahashi et al. (2025)), similar to the practices already implemented in platforms such as GBIF (Telenius 2011), eBird (Tang et al. 2021), and NCBI (Jenuth 1999). By adopting such measures, eDNA can truly deliver valuable data and insights, contributing meaningfully to global biodiversity monitoring and assessment efforts (Altermatt et al. 2025; Thomsen et al. 2024).

## Author Contributions

Y.Z. and F.A. conceived and led the study. Y.Z. collected and analysed the datasets, and wrote a first version of the manuscript, with input and mentoring of F.A. and X.Z. H.Z. assisted with data cleaning. All other authors contributed data and all authors commented on drafts of the paper.

## Funding

This work was supported by National Key Research and Development Program of China, 2022YFC32021001, 2021YFC3201003; National Natural Science Foundation of China, 42507382; Schweizerischer Nationalfonds zur Förderung der Wissenschaftlichen Forschung, 310030_197410, 31003A_173074.

## Conflicts of Interest

X.Z. directs a translation project at Nanjing University that develops apparatus for routine eDNA biomonitoring. The remaining authors declare no competing interests.

## Supporting information


**Figure S1:** Workflow of dataset compilation and reanalysis.
**Figure S2:** Correlation between average sequencing depth per sample and median fish reads number. The blue line represents the generalized linear model (glm) fit with a 95% confidence interval (grey shaded area).
**Figure S3:** Methodological and geographical representativeness of datasets with raw sequencing data (reanalyzable).
**Figure S4:** The barcode reference database coverage of local species across different spatial scales between barcode regions. The local species were defined at the basin, country, and continent scales. The left panels illustrate the genus‐level coverage, while the right panels show the species‐level coverage. The differences between barcodes were assessed by Kruskal‐Wallis rank sum test.
**Figure S5:** The barcode database coverage of local species across different spatial scales between primer sets. The local species were defined at the basin, country, and continent scales. The left panels illustrate the genus‐level coverage, while the right panels show the species‐level coverage.
**Figure S6:** Barcode reference database coverage of the local species list, and the proportion of taxa assigned as local using global assignments, both at genus level, across different spatial scales for each dataset. Local species were defined at the basin (a, d), country (b, e), and continent (c, f) scales. Panels (a–c) show coverage, defined as the proportion of genera in the local species list that were represented in the barcode reference database. Panels (d–f) show the proportion of genera in the global assignment results that were present in the corresponding local species list. The differences between continents were assessed by ANOVA and subsequent pairwise Dunn's tests. Different letters indicate statistically significant differences between groups (p < 0.05). Groups sharing the same letter are not significantly different, whereas groups with no letters in common differ significantly. The horizontal dotted lines indicate a value of 0.5.
**Figure S7:** Proportion of species assigned as local taxa at global assignments. The local species were defined at the basin, country, and continent scales. Left panels illustrate the genus‐level comparisons, while the right panels show the species‐level comparisons. The differences between continents were assessed by Kruskal‐Wallis rank sum test.
**Figure S8:** Comparison of species identity between the original and reanalyzed assignments. The reanalyzed assignments only included species historically recorded at the basin scale.
**Figure S9:** Proportion of sequence reads of species found in both the reanalyzed and the original data set. This figure displays the proportion of sequences corresponding to species that were shared between the original and reanalyzed assignments, with species colour‐coded by continent. The x‐axis represents individual dataset IDs and the y‐axis shows the proportion of sequences (percentage) for shared species between the two assignment methods. Assignments were classified based on either a global or a custom reference database.
**Figure S10:** The global assignment summary for D054_RS048. The category “No_vert” refers to ASVs that were classified as non‐vertebrate taxa.
**Figure S11:** Impacts of fish reads number per sample and sampling year on the proportion of sequencing reads assigned to shared species between reanalyzed and original assignments. Panels (a) and (b) show the effects of the median sequencing depth of datasets (a) and the year of sampling (b), respectively. In both panels, the colour of the data points represents different continents. The regression lines, based on a generalized linear model, illustrate the trends, with the shaded areas representing 95% confidence intervals.
**Figure S12:** Differences in sequence proportion and relative abundance between shared species and species unique to reanalyzed assignments. Panels a–b display the differences in the sequence number proportion between shared species and species unique to the reanalyzed assignments, while panels c–d display the average relative abundance in samples. The x‐axis represents the dataset IDs, distinguishing between global and custom reference databases. Blue boxplots represent the shared species, while orange boxplots represent the species unique to the reanalyzed assignments. The differences between shared species and species unique to reanalyzed assignments were tested for significance using the Wilcoxon test. Comparisons were performed only for datasets that contained both an original species list and a reanalyzed species list, with at least two species in each category (shared and unique to the reanalyzed assignments). Significance levels are indicated as follows: p < 0.001 ***, p < 0.01 **, p < 0.05 *, and p ≥ 0.05 ns.

**Table S1:** Metadata of the 58 studies.
**Table S2:** Parameters used to curate reference database for each primer set.
**Table S3:** Results of normality tests and variance homogeneity checks.
**Table S4:** Fish taxonomy re‐check for datasets from Switzerland (Europe), French Guiana (South America), the St. Regis River (North America), and the Chao Phraya River (Thailand).
**Table S5:** Species list included in the curated reference database for each primer set.
**Table S6:** Results of the generalized linear mixed model showed in Figure 4.
**Table S7:** Results of PERMANOVA and ANOSIM analyzes examining the effects of continents and species assignment types on community composition.
**Table S8:** Significance tests for shared species and number of extra local species between continents showed in Figure S8.
**Table S9:** Significance tests for shared species and number of extra local species between datasets using global or custom reference database in the original assignments showed in Figure S8.
**Table S10:** Summary of reads number for each dataset in the clean ASV table (including all ASVs with or without assignment to vertebrates).
**Table S11:** Results of generalized linear models (GLMs) testing factors influencing the proportion of shared species between original and reanalyzed assignments and the proportion of sequencing reads assigned to shared species.
**Table S12:** Factors influencing the differences in the sequence number proportion and the average relative abundance in samples between shared species and species unique to the reanalyzed assignments.

## Data Availability

The global riverine fish eDNA datasets have been deposited in Dryad under the DOI 10.5061/dryad.gf1vhhn2h and are currently available under private peer‐review status. The data and codes supporting this study are available at https://github.com/Laura61616/GRiversFisheDNA_Tech.
