# Supplementary material for: Integrated Reanalysis of Global Riverine Fish eDNA Datasets Shows Robustness and Congruence of Biodiversity Conclusions
Source: Mol Ecol. 2026 Apr 17;35(8):e70340. doi: 10.1111/mec.70340 (PMC13090749; doi:10.1111/mec.70340)
Supplement: Supplementary file 1 — Figure S1: Workflow of dataset compilation and reanalysis. Figure S2: Correlation between average sequencing depth per sample and median fish reads number. The blue line represents the generalized linear model (glm) fit with a 95% confidence interval (grey shaded area). Figure S3: Methodological and geographical representativeness of datasets with raw sequencing data (reanalyzable). Figure S4: The barcode reference database coverage of local species across different spatial scales between barcode regions. The local species were defined at the basin, country, and continent scales. The left panels illustrate the genus‐level coverage, while the right panels show the species‐level coverage. The differences between barcodes were assessed by Kruskal‐Wallis rank sum test. Figure S5: The barcode database coverage of local species across different spatial scales between primer sets. The local species were defined at the basin, country, and continent scales. The left panels illustrate the genus‐level coverage, while the right panels show the species‐level coverage. Figure S6: Barcode reference database coverage of the local species list, and the proportion of taxa assigned as local using global assignments, both at genus level, across different spatial scales for each dataset. Local species were defined at the basin (a, d), country (b, e), and continent (c, f) scales. Panels (a–c) show coverage, defined as the proportion of genera in the local species list that were represented in the barcode reference database. Panels (d–f) show the proportion of genera in the global assignment results that were present in the corresponding local species list. The differences between continents were assessed by ANOVA and subsequent pairwise Dunn's tests. Different letters indicate statistically significant differences between groups (p < 0.05). Groups sharing the same letter are not significantly different, whereas groups with no letters in common differ significantly. The horizontal dotted [file MEC-35-e70340-s001.docx]

# Supplementary Materials for

**Integrated reanalysis of global riverine fish eDNA datasets shows robustness and congruence of biodiversity conclusions**

Yan Zhang *et al.*

*Corresponding authors: Florian Altermatt: florian.altermatt@uzh.ch and Xiaowei Zhang, zhangxw@ynu.edu.cn

**This file includes:**

Supplementary Text

Captions for Supplementary tables *(Please find the tables in the spreadsheet)*

Supplementary figures

## Supplementary Text

***Complete query used to search the Web of Science Core Collection***

TS = (*monitor* OR *assessment* OR communit* OR richness) AND

TS = (molecular OR "environmental DNA" OR eDNA OR metabarcoding OR "high throughput sequencing" OR "high-throughput sequencing" OR HTS OR "next generation sequencing" OR "next-generation sequencing" OR NGS) AND

TS = (stream$ OR river$ OR estuar*) AND

TS = (fish OR Actinopteri*) AND

PY=(2010-2025) NOT

TS=(qPCR OR ddPCR) NOT

TI=(soil OR diet OR dietary)

TS stands for topic (i.e., title, abstract and keywords), TI for title and PY for publication year. The star character (*) is a wildcard meaning either zero or more characters. The dollar sign ($) means either zero or one character.

***Validation of global species assignments***

The validation of global species assignments using seven datasets with published historical fish records revealed notable differences in geographical scale and taxonomic resolution (Supplementary Table 4). At the basin scale, 74.5% to 100% of species-level assignments were validated as local. In contrast, the validation of genus-level assignments at the same scale exhibited greater variability. For the two datasets from Switzerland, 60% and 100% of assignments were validated as local genera, respectively. Contrastingly, datasets from French Guiana, St. Regis River, and Thailand showed much lower validation rates, ranging from 0% to 11.1%. Although historical records allowed for the validation of some species or genera at the country or continent level, these taxa were not considered local at the basin scale, underscoring the necessity of basin-level data for accurate validation.

## Supplementary Tables

Please find the respective tables in the separately provided spreadsheet.

Supplementary Table 1 Metadata of the 58 studies.

Supplementary Table 2 Parameters used to curate reference database for each primer set.

Supplementary Table 3 Results of normality tests and variance homogeneity checks.

Supplementary Table 4 Fish taxonomy re-check for datasets from Switzerland (Europe), French Guiana (South America), the St. Regis River (North America), and the Chao Phraya River (Thailand).

Supplementary Table 5 Species list included in the curated reference database for each primer set.

Supplementary Table 6 Results of the generalized linear mixed model showed in Figure 4.

Supplementary Table 7 Results of PERMANOVA and ANOSIM analyzes examining the effects of continents and species assignment types on community composition.

Supplementary Table 8 Significance tests for shared species and number of extra local species between continents showed in Supplementary Figure 8.

Supplementary Table 9 Significance tests for shared species and number of extra local species between datasets using global or custom reference database in the original assignments showed in Supplementary Figure 8.

Supplementary Table 10 Summary of reads number for each dataset in the clean ASV table (including all ASVs with or without assignment to vertebrates).

Supplementary Table 11 Results of generalized linear models (GLMs) testing factors influencing the proportion of shared species between original and reanalyzed assignments and the proportion of sequencing reads assigned to shared species.

Supplementary Table 12 Factors influencing the differences in the sequence number proportion and the average relative abundance in samples between shared species and species unique to the reanalyzed assignments.

## Supplementary Figures


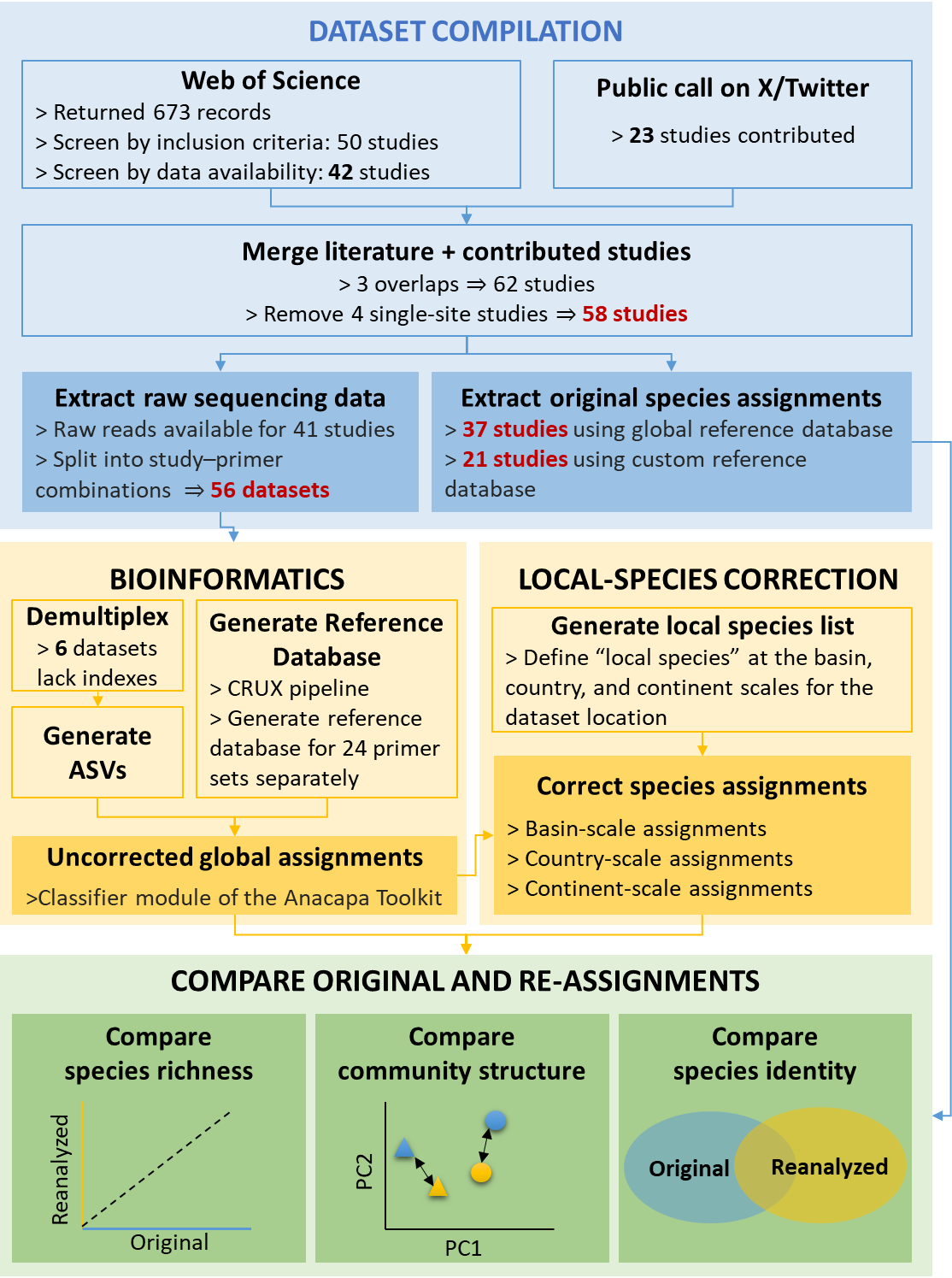


Supplementary Figure 1 Workflow of dataset compilation and reanalysis.


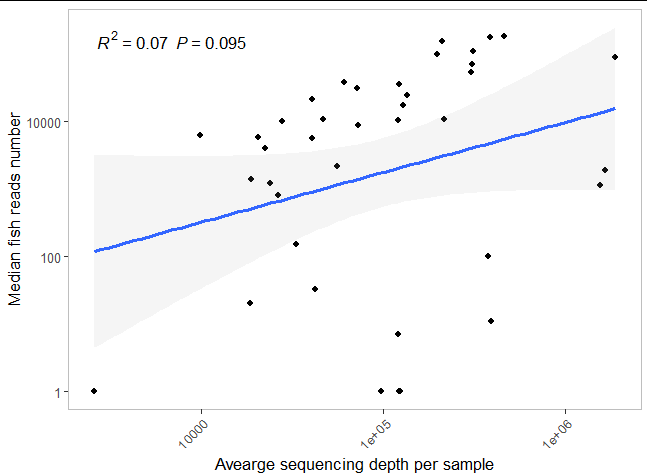


Supplementary Figure 2 Correlation between average sequencing depth per sample and median fish reads number. The blue line represents the generalized linear model (glm) fit with a 95% confidence interval (grey shaded area).


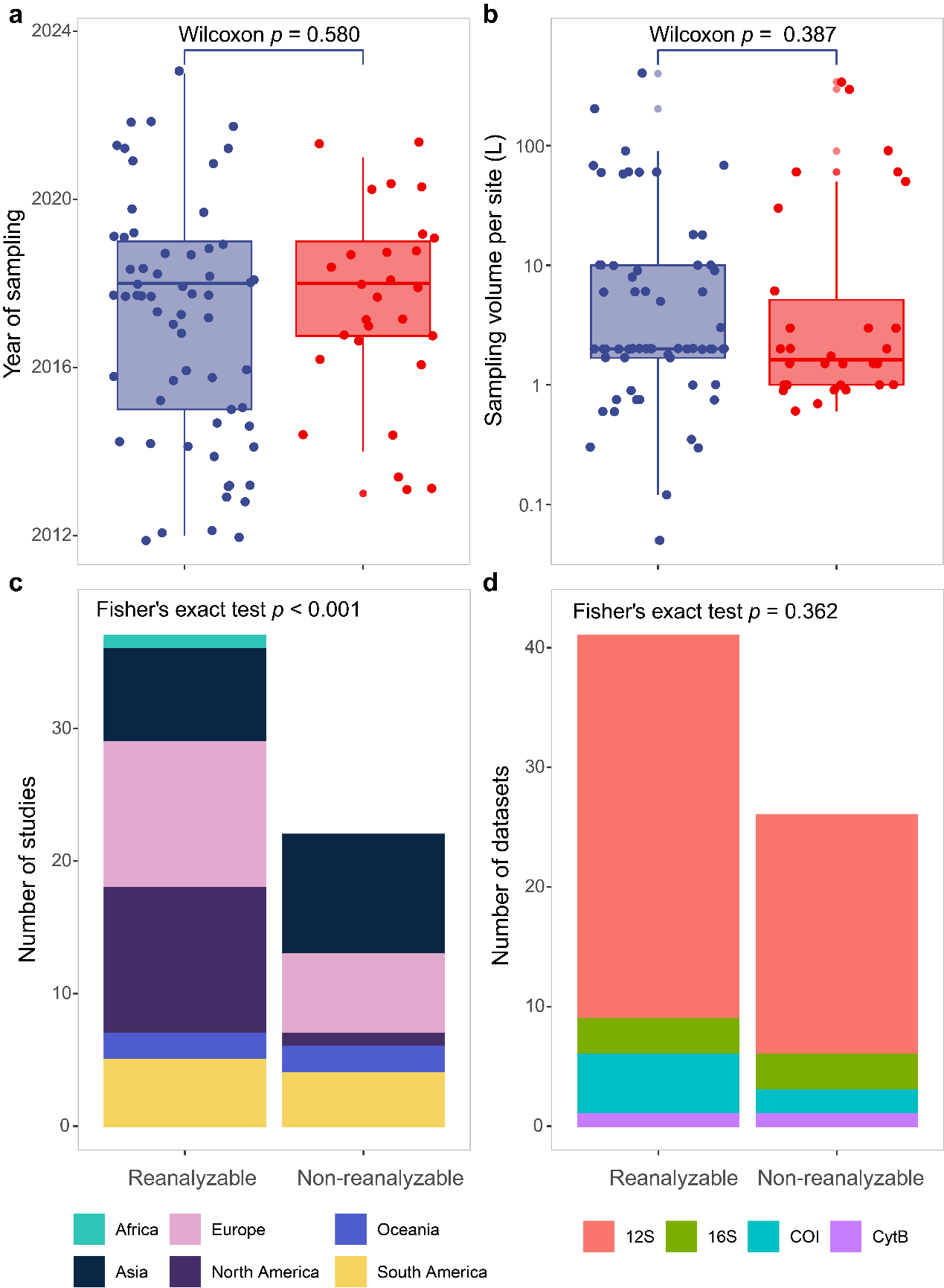


Supplementary Figure 3 Methodological and geographical representativeness of datasets with raw sequencing data (reanalyzable).


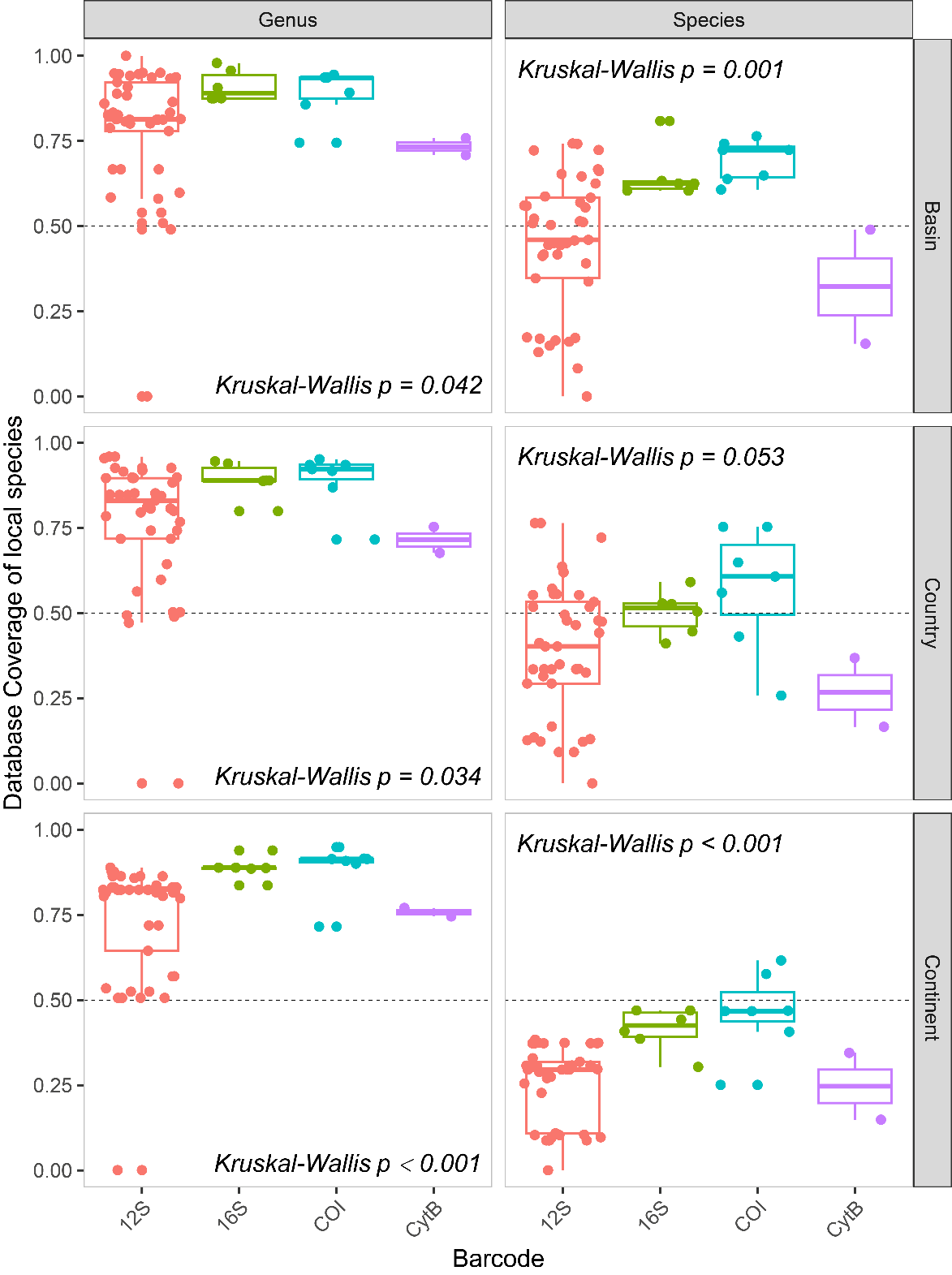


Supplementary Figure 4 The barcode reference database coverage of local species across different spatial scales between barcode regions. The local species were defined at the basin, country, and continent scales. The left panels illustrate the genus-level coverage, while the right panels show the species-level coverage. The differences between barcodes were assessed by Kruskal-Wallis rank sum test.


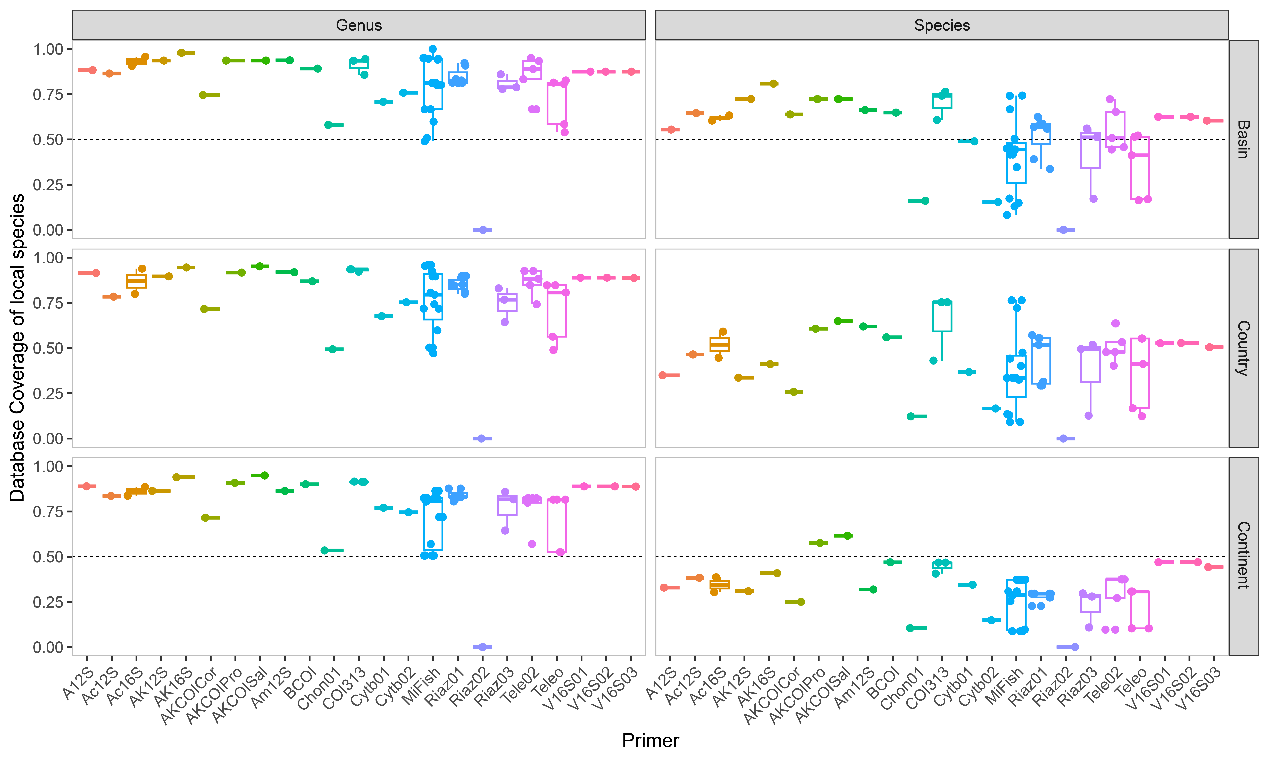


Supplementary Figure 5 The barcode database coverage of local species across different spatial scales between primer sets. The local species were defined at the basin, country, and continent scales. The left panels illustrate the genus-level coverage, while the right panels show the species-level coverage.


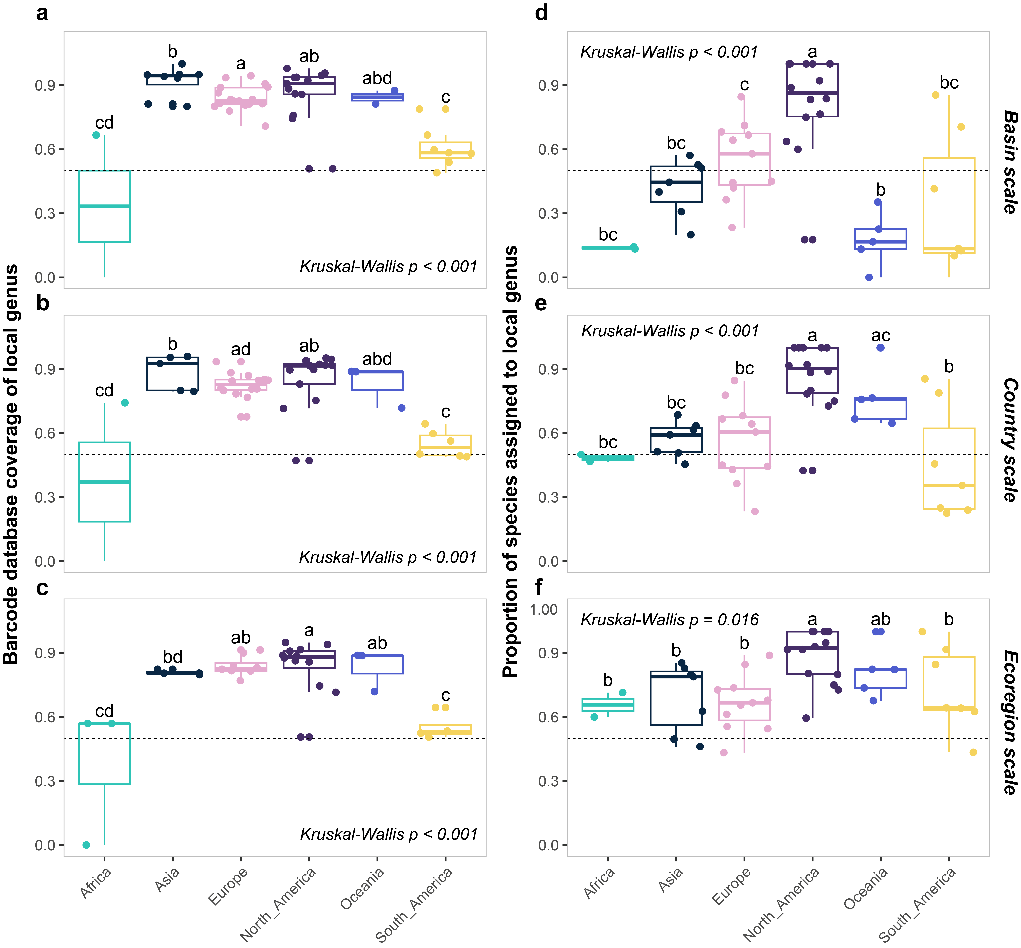


Supplementary Figure 6 Barcode reference database coverage of the local species list, and the proportion of taxa assigned as local using global assignments, both at genus level, across different spatial scales for each dataset. *Local species were defined at the basin (a, d), country (b, e), and continent (c, f) scales. Panels (a–c) show coverage, defined as the proportion of genera in the local species list that were represented in the barcode reference database. Panels (d–f) show the proportion of genera in the global assignment results that were present in the corresponding local species list. The differences between continents were assessed by ANOVA and subsequent pairwise Dunn’s tests. Different letters indicate statistically significant differences between groups (p < 0.05). Groups sharing the same letter are not significantly different, whereas groups with no letters in common differ significantly. The horizontal dotted lines indicate a value of 0.5.*


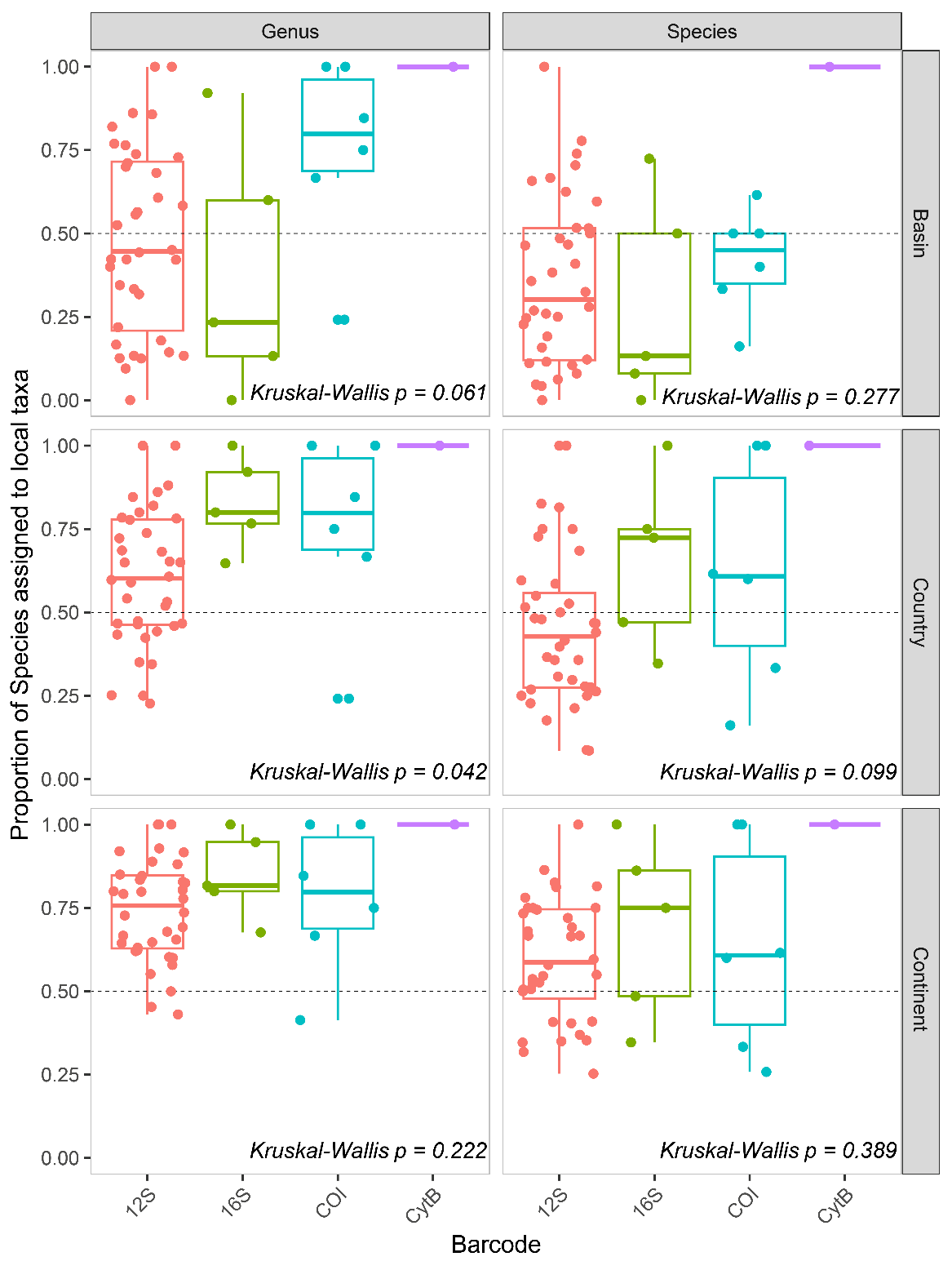


Supplementary Figure 7 Proportion of species assigned as local taxa at global assignments. The local species were defined at the basin, country, and continent scales. Left panels illustrate the genus-level comparisons, while the right panels show the species-level comparisons. The differences between continents were assessed by Kruskal-Wallis rank sum test.


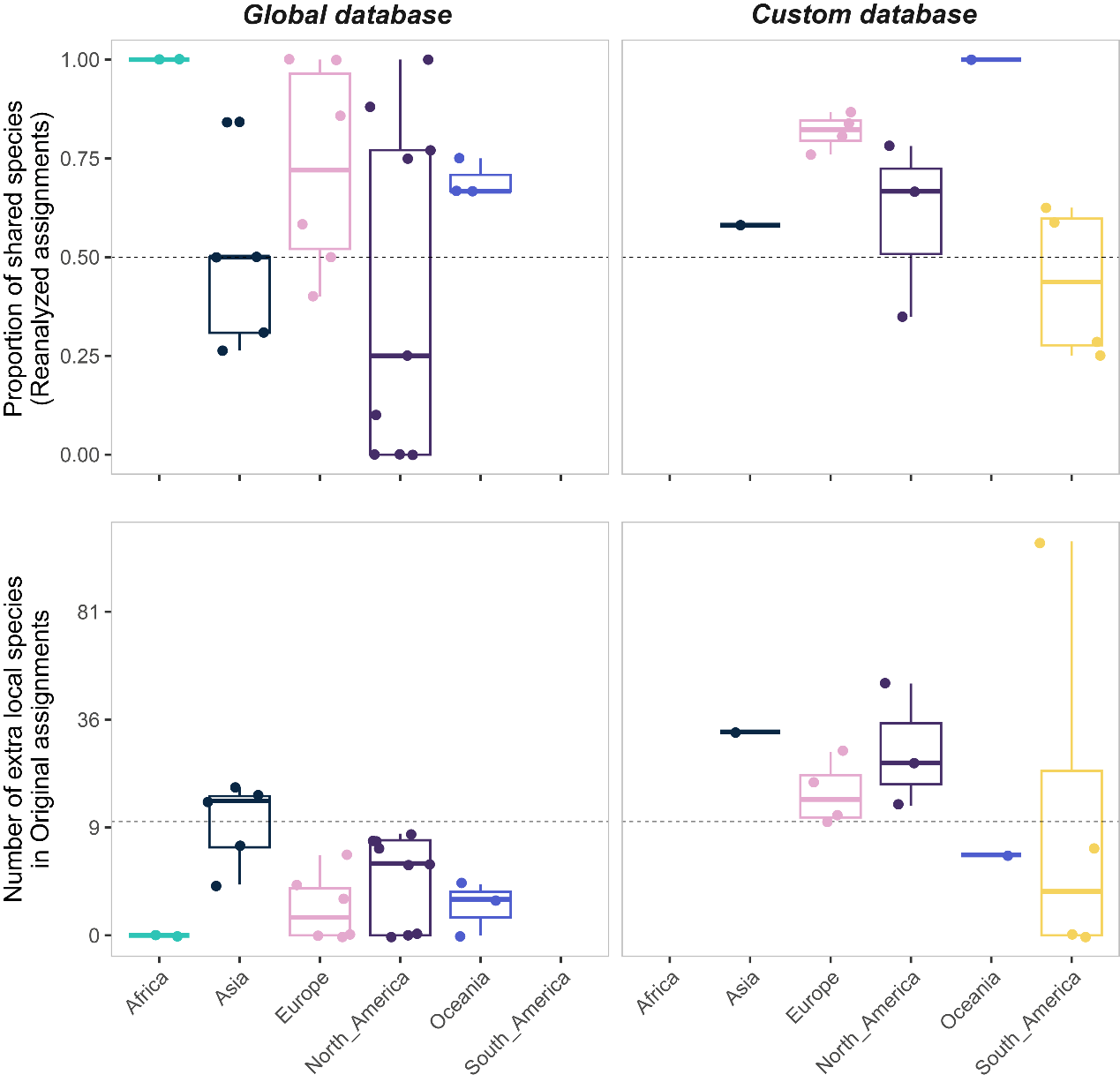


Supplementary Figure 8 Comparison of species identity between the original and reanalyzed assignments. The reanalyzed assignments only included species historically recorded at the basin scale.


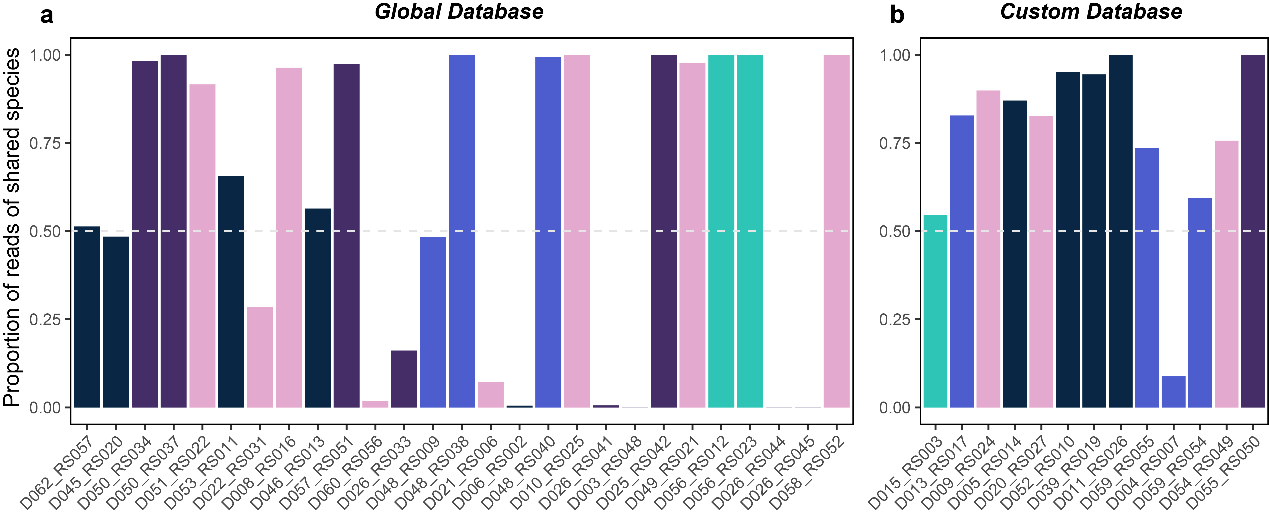


Supplementary Figure 9 Proportion of sequence reads of species found in both the reanalyzed and the original data set. This figure displays the proportion of sequences corresponding to species that were shared between the original and reanalyzed assignments, with species color-coded by continent. The x-axis represents individual dataset IDs and the y-axis shows the proportion of sequences (percentage) for shared species between the two assignment methods. Assignments were classified based on either a global or a custom reference database.


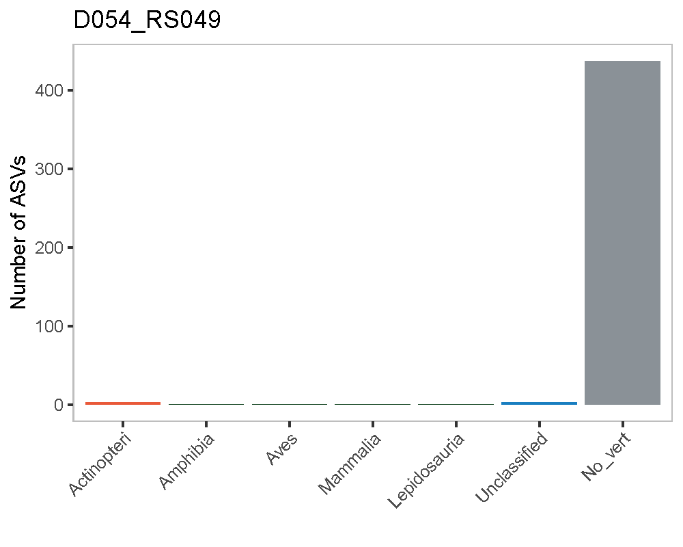


Supplementary Figure 10 The global assignment summary for D054_RS048. The category “No_vert” refers to ASVs that were classified as non-vertebrate taxa.


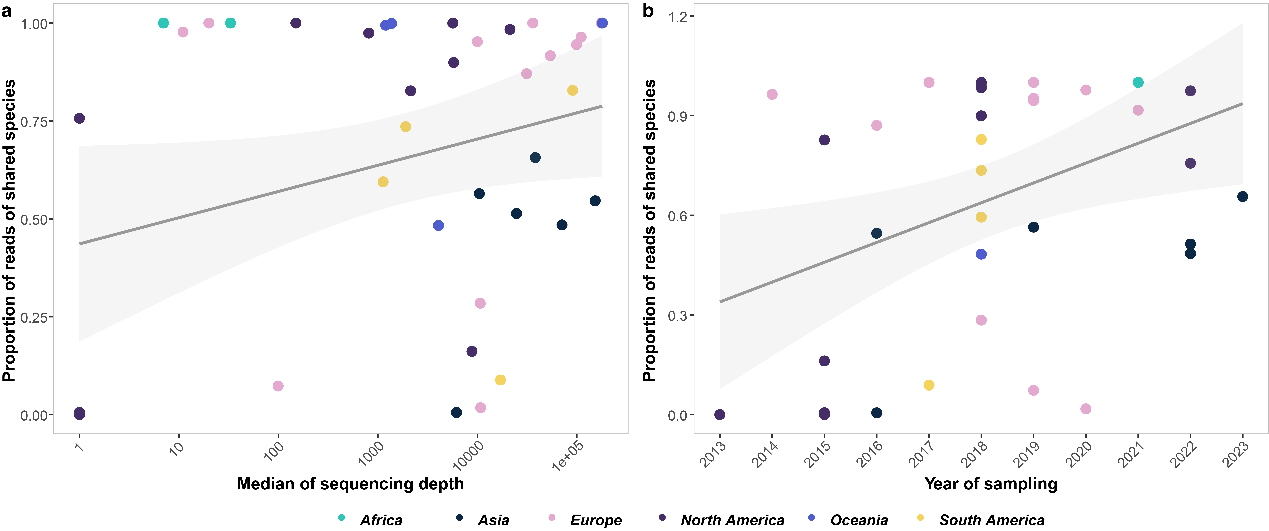


***Supplementary Figure 11 Impacts of fish reads number per sample and sampling year on the proportion of sequencing reads assigned to shared species between reanalyzed and original assignments.*** *Panels (a) and (b) show the effects of the median sequencing depth of datasets (a) and the year of sampling (b), respectively. In both panels, the color of the data points represents different continents. The regression lines, based on a generalized linear model, illustrate the trends, with the shaded areas representing 95% confidence intervals.*


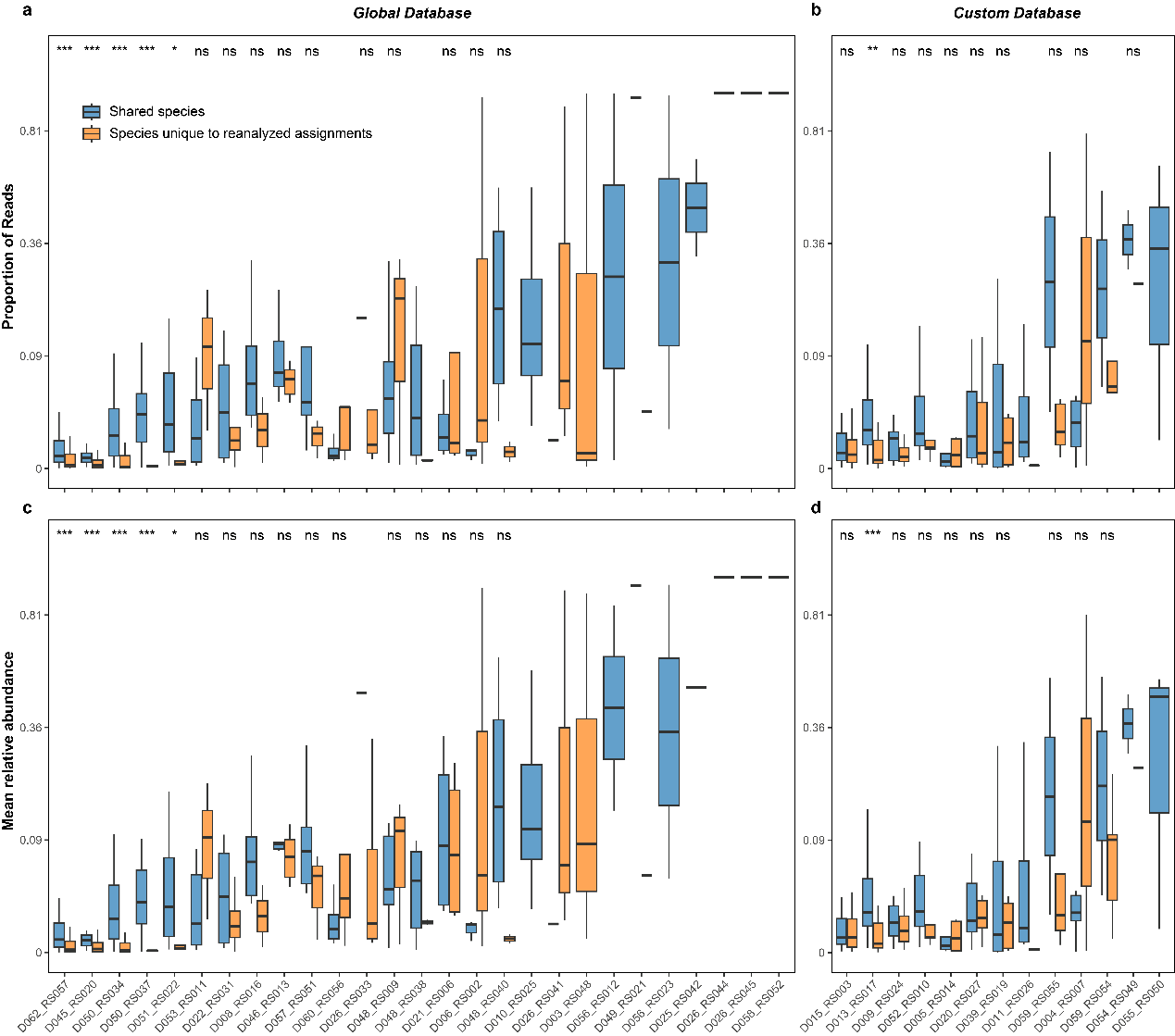


Supplementary Figure 12 Differences in sequence proportion and relative abundance between shared species and species unique to reanalyzed assignments. Panels a–b display the differences in the sequence number proportion between shared species and species unique to the reanalyzed assignments, while panels c–d display the average relative abundance in samples. The x-axis represents the dataset IDs, distinguishing between global and custom reference databases. Blue boxplots represent the shared species, while orange boxplots represent the species unique to the reanalyzed assignments. The differences between shared species and species unique to reanalyzed assignments were tested for significance using the Wilcoxon test. Comparisons were performed only for datasets that contained both an original species list and a reanalyzed species list, with at least two species in each category (shared and unique to the reanalyzed assignments). Significance levels are indicated as follows: p < 0.001 ***, p < 0.01 **, p < 0.05 *, and p ≥ 0.05 ns.
